# Clinic of the Jefferson Medical College

**Published:** 1851-03

**Authors:** 


### CLINICAL REPORTS.

*Clinic of the Jefferson Medical College.* Service of Profs. PANCOAST and MÜTTER.

Number of cases treated, from Sept. 1st to Dec. 21st, as reported, 906 ; operations 120.

*Cases treated from Dec. 21st, to Jan. 18th.*

Whole number of cases, 279 ; operations, 24, viz. :

Cancer of the Breast, 1 ; Hydrocele, (removal of a portion of the tunica vaginalis,) 1 ; Strabismus 2 ; Onychia 1 ; Nasal Polypus, 1; Fistula Lachrymalis, 2; Hæmorrhoids, 2; Pterygium, 1 ; Ectropion, 1 ; Enlarged Tonsils, 2 ; Elongated Uvula, 1 ; Fatty Tumor of Scapula, 1 ; Prolapsus Iridis, 1 ; Varicose Veins, 1 ; Polypous Tumor of eye-lid, 1; Hygroma of knee, 1 ; Removal of a musket ball and several buck-shot from the thigh, 1 ; Ganglion of Wrist, 1 ; Section of the Pectineus, 1 ; Cataract—lens displaced with the needle. Days of operating, 7 ; there being no clinic on Dec. 25th, and 28th. Average number of patients each day, 39.8.

*Clinic of October 9th,* 1850.

*Cases presented.*—Fistula Lachrymalis ; Ulcer of Ankle ; Retention of Testicles ; Osteitis, giving rise to bending of tibia ; Scrofulosis ; Tumor of back ; Wound of hand from fractured glass ; Acute Osteitis ; Strabismus ; Scrofulous Ophthalmia ; Polypus of nose ; Hypopion ; Steatomatous Tumor on arm ; Aneurism of Aorta ; Pseudarthrosis of humerus, seton introduced between the ends of the bone ; Two great toes on each foot, amputation ; Caries of Femur ; Medullary Sarcoma ; Caries of Spine ; Tumor on Cheek ; Abscess on side of Face, opened ; extensive Cicatrix from burn of arm, producing great deformity ; Chronic conjunctivitis ; Amaurosis ; Lithotomy ; Total, 28.

CASE 28.—URINARY CALCULUS IN THE BLADDER.

Professor PANCOAST said : The next case to which I shall call your attention, is one of stone in the bladder.

The patient, Mr. J. Messersmith of Lehigh Co., in this State, is one of our upright and respectable Pennsylvania farmers. He is about 56 years of age, of a robust frame, as you may see, easy in circumstances, and has always led, as I learn from his physician, Dr. Hoffman, who was good enough to direct him to me, a very moral and temperate life.

He comes from beyond the lime-stone region of this State, a fact of some interest, as connected with the origin of the stone. He has been suffering for several years with the ordinary symptoms of urinary calculus, and for a few months past has discharged a good deal of blood with his urine. During fits of the stone, brought on, as they usually have been, after walking or riding. the quantity discharged has amounted to a hemorrhage. On this account, partly, and partly on account of the absence of any symptoms indicative of disease of the kidney, we are at once led to infer that the source of the hemorrhage is the mucous membrane of the bladder. This inference is converted into positive proof by the effect of sounding the bladder, for this, no matter how delicately it is done, is followed by bleeding.

On examination by the rectum, I find the prostate gland hypertrophied and large ; by use of the sound, I discover that the middle lobe is large and soft, and that there is a deep pouch or bas-fond to the bladder beneath it, in which the stone is lodged. The urethra is apparently healthy.

The stone is a hard one, as it rings when struck with the sound. It lies loose and unattached, as I know from being able to pass the sound between it and the walls of the bladder. I have picked it up with the lithontriptor, and measured its dimensions by the scale which is attached to the outer end of the instrument. If there be but a single stone, it is about an inch long and five-eighths of an inch thick—as by dropping and picking up the stone again in the bladder, I have ascertained these two measurements. It is possible, however, that there may be two calculi, which I have gotten hold of separately. The bladder, notwithstanding the disposition of its neck to bleed, bears pretty well a moderate degree of distention, as I have injected it with six ounces of warm water for the purpose of sounding, a preparatory step of much importance both as regards sounding for the detection of the stone, and the use of the lithontriptic instrument, if we should decide upon crushing the stone in the bladder.

Now, gentlemen, you have the case fully before you, and the patient is at hand, willing to submit to any operation that will restore him the most completely to a state of health. Shall we crush the stone in its place, which I can do without difficulty, and let the fragments be gradually discharged with the urine, or shall we cut him and extract the stone at once? This is a case unlike most that present themselves, where either operation would really be allowable. But in most cases of stone, to a surgeon equally familiar with both plans of operation, one or the other is clearly indicated as the best.

Now you all know, at least such as are attending their second or third course in this institution, the general preference, as suited to the greater number of cases, that I give to the crushing over the cutting operation, on the grand account of its being attended with less hazard to life. Yet it has so happened during the course of the last three winters, that you have seen me operate in this place on six separate cases of stone—three by the cutting and three by the crushing process, and now I have decided on cutting a fourth. Of those cut, two were young boys, one a man of thirty, and this is, as you see, a man passed middle life.

Of the three men subjected to the crushing process, one was twenty-five and the others fifty-eight and seventy years of age. I make these details, lest you should be led astray,—this preponderance of the cases cut over those crushed, being a mere matter of accident; for in private practice I crush four or five times at least, where I cut once. There are rules, however, by which we should determine the fitness of each plan of operation to particular cases, and I will briefly mention such as influence me in the choice.

1st. As regards females. You should cut no female except in a case of urgent necessity. As a general rule the stone is to be broken up in the female bladder; or if it be small, you have the choice instead of breaking it to dilate the short distensible urethra, and withdraw the stone with a small pair of forceps.

I have succeeded without difficulty in breaking up calculi of the hardest kind in females, for the urethra will admit of the employment of strong and powerful instruments for that purpose. You will see the debris, the result of many of these operations, in the museum. Larger stones may be more readily gotten rid of by crushing in the female bladder than in the male, as there is no deep bas-fond, and no prostate gland to become enlarged, fungous and irritable. You may observe in the museum the fragments of a large stone, which was two and a-half inches in in its long diameter, which I removed many years ago with the most complete success, from a lady at Emerysville, in Chester county, about fifty years of age. It required, however, thirteen successive operations to reduce it to fragments of such size as to be discharged with the urine.

The size of the calculi, therefore, does not influence the choice of operation in the female. They are to be crushed, and I have succeeded in crushing them of almost all sizes. The exceptional cases in the female, in which cutting is allowable, it appears to me, are those in which the stone is of moderate size, and jammed in at the inner end of the urethra so as not to be readily pushed back, or when a rough stone of considerable dimensions has excited so much irritation and thickness of the coats of the bladder as to make that organ utterly intolerant of such a degree of distention by the injection of fluids, as to enable us to open and shut the lithontriptic instrument without injury to its coat. In two cases of this sort I have, however, succeeded in getting a sufficient degree of toleration for the use of the instrument, by injecting two or three ounces of sweet oil instead of water or mucilage.

In two cases of females I have been obliged to perform lithotomy. One was in a child, a case of impacted stone. The other was that of a girl of nineteen, a patient of Dr. Knorr, of this city, who suffered intensely from a stone one inch and three-eighths in diameter, tightly embraced by an intolerant bladder. In both cases I cut from the urethra upwards, towards the symphysis pubis, and closed the sides of the incision as well as I could, after the extraction of the stone, with sutures introduced along the urethra. In both instances I found it necessary to retain an open catheter in the urethra, to prevent the blood, accruing from the wound in the neck, being drawn by the suction of the respiratory movements back into the cavity of the bladder.

The liability to have incontinence of urine follow these operations in the female is the great and serious objection to their performance, and I believe there is no way, if the stone be extracted by an incision below the arch of the pubis, in which it can with any certainty be avoided. The child suffered from this cause to a considerable degree. The older patient escaped with the power of retaining the urine readily for four or five hours, and the escape appeared to me to be owing to the careful closure of the line of the urethral incision by the interrupted suture.

You will perceive from these views of mine, that I am disposed to exclude the high or super-pubic operation which is so commonly recommended in females. For if the bladder is dilatable, I have always been able to crush the calculus contained within it; and if it is not dilatable, the operation above the pubis cannot be resorted to without an unjustifiable risk of injury to the peritoneum.

2nd. In males; the first consideration is the question of age. I believe in children of this sex, it is proper almost uniformly to perform lithotomy.

*a.* As regards the urethra. It is said by most writers, that stricture in the urethra, great irritability, or even an undilatable condition of the urethra, furnish sufficient cause for the preference of the cutting over the lithontriptic operation. But I have not found it so; I have met with all these obstacles and been able to overcome them. Strictures, if they will not otherwise yield, I divide with the instrument of Dr. Dodd, and then dilate them. If the irritability or undilatability of the urinary canal is not soothed by the gentle use of dilating instruments, by the frequent use of the warm bath, careful attention to regimen and the melioration of the urine by the use of internal remedies, I add to these measures, a slight cauterization of the irritated part with the porte-caustic of Lallemand, and then after a short interval revert again to their use.

*b.* The internal organs. Upon the condition of the prostate, the bladder and the kidneys, from the age of puberty upwards, should I believe, invariably depend the choice of the kind of operation to be practised. It is therefore necessary to determine their state as accurately as possible beforehand.

If the kidney be seriously involved, and especially if the symptoms indicate a suppuration of its pelvis on either side, you are in no wise to cut, for the system is too seriously undermined to withstand the shock of the operation and the protracted suffering of the cure. The operation by crushing, is the only alternative, if it be proper to operate at all.

If the bladder be diseased in its mucous coat merely, although freely throwing out mucus, muco-purulent matter, or pus, or acting upon the urine so as to cause a deposit of white gravel on its lining membrane, with the prostate and neck in a tolerably healthy condition, and capable of bearing for a few minutes without much suffering to the patient, the injection of an half a pint or more of warm water or mucilage, and the stone be not excessively hard, it is I believe your bounden duty in all cases to operate by the crushing process. Before and during the course of treatment, general and local measures, such as baths and tonics, acids or alkalies according to the chemical nature of the urine, and the condition of the bladder, may be employed with advantage.

But if the bladder be in a condition such as that first described, or in a worse condition even, so that it will not bear distension, either from the local accumulation of urine, or by injection from without; hypertrophied as to its muscular coat; sensitive to the contact of instruments, and easily excited to contractions which will grasp the sound by its walls; ribbed or columnar, as you may tell by passing the sound over it; or sacculated as you may sometimes discover by the point of the sound falling into recesses—the condition of the prostate, it appears to me, is then alone to determine whether you are to cut for, or crush the calculus. On this subject, which has given rise to much conflict of opinion among writers, I entertain somewhat peculiar opinions, the result of a good deal of experience and careful observation. If the prostate be healthy, firm, and but little or not at all enlarged, it is, I think the duty of the operator to crush; but if it is enlarged generally, and especially in its middle lobe, and as is commonly the case under these circumstances, bleeding after the use of the sound, and with a deep bas-fond behind it, it is I believe better to cut. It might seem to you that the large size of the prostate gland should offer a counterindication to lithotomy, and render lithontripsy the more eligible operation. But you must recollect that the large prostate gives a deep bas-fond, and the fragments of the stone will therefore not so readily escape, requiring to be minutely broken—which would necessitate the frequent use of the lithontriptor. The irritation arising from this, and the motion of the fragments in the bladder, will tend to produce so much vesical irritation as to endanger and perhaps to destroy life. If the bladder be sacculated, these fragments by lodging in them might, with their sharp angles, excite ulceration through the walls of the organ. In such cases the operation by crushing is attended with as much, perhaps even more danger, than that of lithotomy, though it is not so immediate, nor so directly traceable to the operation. You will, therefore, occasionally meet with cases of stone in old men with such derangement of the bladder, prostate and kidneys, that it will be most judicious not to attempt either form of operation.

The enlargement of the prostate is, per se, a strong reason with me in favor of lithotomy. It is in itself a disease over which our medicines have but little influence. It is said by Lallemand that the most effectual method of reducing its size and relieving its distressing symptoms, is to divide it freely, and for this object alone he has made the bilateral cut, as in the operation for stone. Of this I am well assured, that I have seen it reduced greatly in size and cease to cause much inconvenience after lithotomy. I show you two calculi of near an inch in their long diameters, which I removed about two months since from an aged gentleman near Mount Holly. In this case I decided to cut in consequence of the large size of the prostate, and made the bi-lateral incision of Dupuytren. The subject was spare, yet so large was the prostate that I could barely, after the incision, reach its top with my finger, and was obliged to use the curved forceps to grasp the stones from the bottom of the bladder. Yet the recovery was most rapid, as the gentleman was riding about the country again in four weeks without the slightest inconvenience on the side of the bladder.

I have now given you briefly, the more prominent reasons that weigh with me in the choice of operation, as applied to particular cases of stone.

Having decided to cut for stone, which of the three routes described in the books shall we select through which to make our entrance into the bladder? Through the linea alba into the front part of the body of the bladder, as in the super-pubic operation; through the rectum and prostate, as in the recto-vesical; or by the perineum?

The first appears to me indicated only in those cases, very rarely met with, in which there is a stone too hard to be broken, and too large to be withdrawn under the arch of the pubis. The second seems to me indicated solely in the exceptional cases where the stone is lodged in the sinus of the prostate, and not susceptible of being pushed back into the bladder. The perineal is, by common consent, the most generally appropriate form of operation, and to this I shall restrict my remarks. Of these we have two forms, both of which require to be studied; the lateral and bi-lateral. In neither of these do we cut the bladder itself, we merely open a way into it by cutting through the prostate. This organ, if it be healthy, is very distensible, and requires an incision of but little width to allow of the withdrawal of a calculus of moderate dimensions, and if it be enlarged and hard, it admits of a wide incision.

In the lateral operation we cut one lobe of the prostate merely, and the left, because it is most convenient to use the right hand.

In the bi-lateral we divide both lobes. Each of these are employed by different operators as the exclusive mode, for all ages and all states of the organs. But each one has its peculiar fitness for particular cases. The lateral is most speedily done and is the most appropriate when you have a healthy prostate, a thin perineum, and a small stone. It is especially to be preferred in cases of children, where the prostate is yet rudimental.

To perform this operation I require but three instruments: a curved staff, with a deep groove on its convex part; an ordinary French scalpel, with a long handle; and a pair of forceps curved downwards near the end of the blades. The anatomy of the parts through which you have to cut you already know, as I have prepared you for the operation of to-day, by demonstrating the structure of the perineum in an evening lecture. I distend the bladder moderately with water, introduce the staff and touch the stone; my object is then to open the membranous part of the urethra, without cutting the bulb, the improper division of which is the common source of arterial hemorrhage after this operation. But two strokes of the knife are required to reach the membranous part of the urethra. The knife should be entered on the raphe of the perineum, about midway between the root of the scrotum and the anus, and drawn down, cutting deeper as it descends, to a point midway between the tuberosity of the ischium and the middle of the anus. The second stroke should be made by entering the knife about five-eighths of an inch below the top of the first, (as this will clear the bulb,) and drawing it down, cutting deeper as we descend, to within the same distance from the lower end of the first incision. By pushing the finger in at the middle of the wound you will now feel the staff below the bulb, covered by the membranous part of the urethra. Sink the nail of the left forefinger into the groove of the staff, conduct the point of the same knife along the nail, cut through the wall of the membranous part of the urethra till the knife grates freely in the groove of the staff; then hold the knife firmly against the staff, with the edge directed diagonally towards the left side. Take, now, the staff from your assistant, with your left hand, draw the handle downwards towards the thighs, and the knife, almost without a feeling of resistance, will be carried into the bladder.

The prostate is now divided, and the water will gush out through the wound. Before withdrawing the knife I raise the handle a little, so as to nick a little deeper the vesical end of the prostate, which is its most unyielding part. Both staff and knife are then to be withdrawn, the finger passed through the wound into the bladder, bent about a little, so as to dilate the cut in the prostate, the forceps conducted along it into the bladder, the stone seized and withdrawn. This is, I am convinced, the neatest, surest, and, what is of less importance, the speediest mode of making the lateral cut for stone.

In the two children whom many of you saw me operate on in this place, the whole operation occupied, in the one case, a little less, and the other a little more, than a minute. The only point in this process different from the common operation performed with the knife, is, that I draw down the handle of the instrument as if I were using the gorget, and let it carry the knife in, which is merely firmly held with a slight pressure forward, up against the curve of the staff. The prostate may be very safely divided by pushing in the gorget a sort of sloping chisel, as you see, or with the single lithotome-caché, which makes the cut as it is withdrawn by its two blades, which have been opened in the bladder.

Both of these instruments, however, require the previous use of the knife to open the membranous part of the urethra.

The bi-lateral operation is that which I shall perform to-day. This is, I believe, by far the most appropriate and least hazardous when the stone is large, the perineum deep and vascular, or the prostate enlarged and hard. You cut by this both lateral lobes of the prostate, drain it of its fluids, so that each lobe may be subsequently expected to undergo a sort of atrophy by which it will be reduced more nearly to its natural healthy dimensions, and you get a free opening directly at the bottom of the bladder, which gives you ready access to the stone, and leaves (a most important matter) a free outlet to the urine, which is a great safeguard against the risk of urinary infiltration and abscess. You rarely divide a vessel that requires a ligature; it is scarcely possible that you could cut beyond the boundaries of the prostate so as to endanger the pelvic fascia, or wound the prostatic plexus of veins which are without valves, and, when cut in old men, bleed copiously, from their direct connection with the vesical plexus; or injure the pudic artery; all of which is more or less likely to happen in the lateral operation, especially under the use of the gorget or single lithotome-caché.

We will now proceed to the operation. The patient will have the bladder moderately distended by the injection of warm water through a catheter, have the staff, which is deeply grooved on the back part, introduced, and inhale ether. The hands and feet will be secured together, as is ordinarily done in these operations, and the pelvis raised on a folded blanket, placed on the end of a narrow table. We have taken the precaution to have the pouch of the rectum emptied, by an injection, so that it may not come in the way and be cut.

When the patient had become insensible under the use of the ether, the operation was commenced by making an incision with the scalpel round the anterior semi-circumference of the anus, a half an inch in depth, and five-eighths of an inch from its margin.

This was deepened again by the use of the knife, in the direction of the membranous part of the urethra, cutting just beneath the transversales perinei muscles, so as not to divide any artery of consequence. After this second use of the knife, the staff could be felt in the membranous part of the urethra; this was then divided over the groove of the staff, and the double lithotome placed with its point in the groove, and passed into the bladder. The staff was then withdrawn, and the lithotome, turned with its concave surface downwards, was drawn out horizontally with its two blades expanded, dividing both lobes of the prostate in its course. The forceps was then introduced, and two calculi of the size above indicated, withdrawn from the bladder. The operation occupied but little more than two minutes, and the patient was scarcely conscious of its performance. There were no arteries to be tied. The patient was then placed in his bed in a recumbent posture. A large sized gum catheter was introduced through the wound, and retained for three days, to ensure a ready escape to the urine. An anodyne and diaphoretic mixture was administered at regular intervals, so as to keep the patient for a couple of days steadily under its soothing influence. No fever, and but little pain or irritation followed. On the fourteenth day after the operation, the wound was so nearly closed, that the patient walked into the clinic, and was presented to the class. In a week more the cicatrization was complete, and in twenty-three days from the time of the operation, he felt himself so completely recovered, as to undertake his journey home, by the stage, a distance of forty miles, which he accomplished in one day, and without any serious inconvenience.

